# Development to implementation to evaluation: step-by-step approach to embedding a clinical trial of a machine learning algorithm in the electronic health record

**DOI:** 10.1093/jamiaopen/ooag180

**Published:** 2026-09-21

**Authors:** Erin Morrison, Jaes Overley, Kaitlin Wurm, Don Mock, Sivasubramanium V Bhavani

**Affiliations:** Emory Healthcare, Atlanta, GA, United States; Emory Healthcare, Atlanta, GA, United States; Emory Healthcare, Atlanta, GA, United States; Emory Healthcare, Atlanta, GA, United States; Department of Medicine, Emory University School of Medicine, Atlanta, GA 30307, United States; Emory Critical Care Center, Atlanta, GA, United States

**Keywords:** Sepsis, AI, EHR, pragmatic trials

## Abstract

**Objective:**

To describe the implementation process for the Precision Resuscitation with Crystalloids in Sepsis (PRECISE) trial, a pragmatic, multihospital randomized controlled trial (RCT) of a machine learning (ML) algorithm integrated into the electronic health record (EHR).

**Materials and Methods:**

Implementing PRECISE involved building 4 key components into the EHR using standard tools within Epic: (1) automated inclusion criteria, (2) real-time subphenotyping. (3) randomization, and (4) medication alternative alert.

**Results:**

The PRECISE trial was deployed across 6 Emory Healthcare hospitals in June 2024, spanning 6 emergency departments and 17 ICUs with more than 300 ICU beds.

**Discussion and Conclusion:**

PRECISE operationalizes an ML algorithm within the Epic to identify a subgroup of sepsis patients and prompts the clinician to order the type of fluids thought to confer a mortality benefit in this subgroup. PRECISE demonstrates that precision RCTs of ML algorithms can be executed entirely within the EHR, a framework that represents a model for future learning health systems.

## Introduction

The Precision Resuscitation with Crystalloids in Sepsis (PRECISE) trial is a pragmatic, multihospital randomized controlled trial (RCT) designed to test a precision medicine approach to fluid resuscitation in sepsis.^1^ Despite intravenous fluids being the most common treatment given to patients with sepsis, it is still unclear whether the type of crystalloid fluids impacts mortality. Decades of trials comparing balanced crystalloids to normal saline have not shown consistent mortality differences in critically ill patients, suggesting that a 1-size-fits-all approach may miss subgroups that may benefit from specific fluids.^2–6^ PRECISE uses a validated machine learning (ML) algorithm that classifies patients into sepsis subphenotypes using bedside vital signs (ie, temperature, heart rate, respiratory rate, systolic, and diastolic blood pressure); 1 subphenotype (Group D) has been shown to have a substantial mortality benefit from balanced crystalloids compared to normal saline in secondary analyses.^7–9^ Group D patients are characterized by normal temperature, heart rate, and respiratory rate, and low systolic, and diastolic blood pressure.

**Figure 1.**
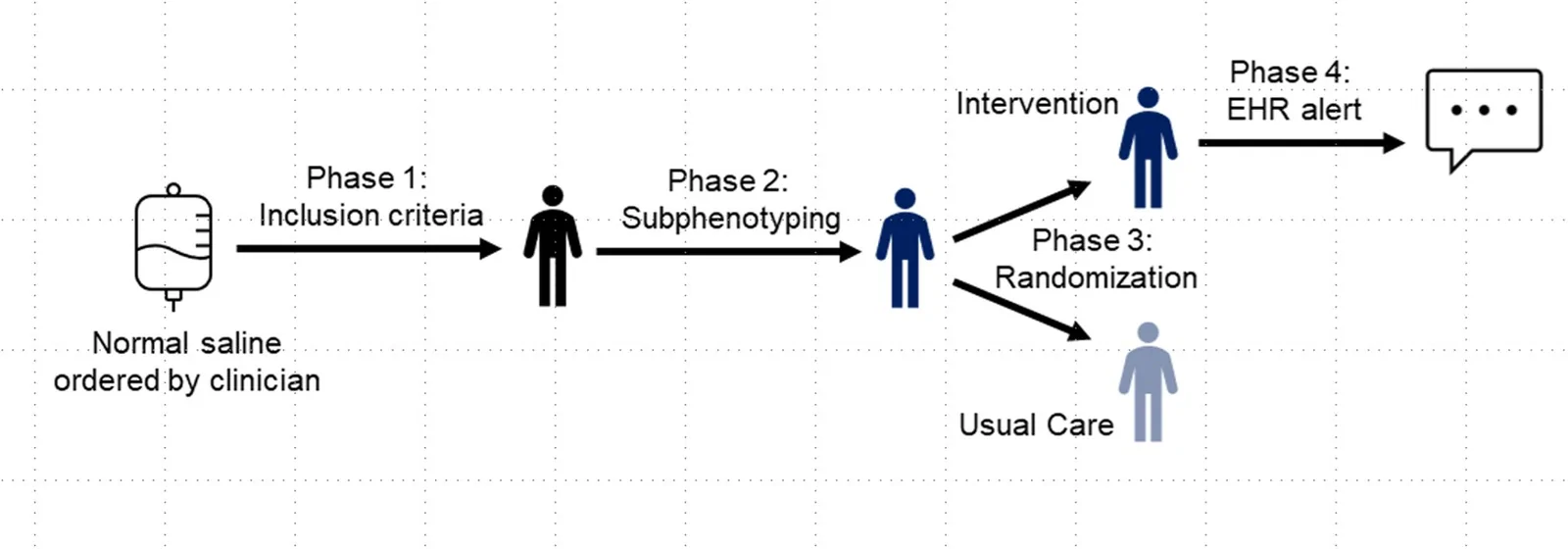
The PRECISE trial is an EHR-integrated pragmatic RCT that enrolls Group D patients and randomizes them to usual care or intervention, with the intervention being an EHR-alert that nudges the clinician to choose balanced crystalloids instead of normal saline.

In the PRECISE trial, patients identified as Group D and meeting inclusion criteria are randomized to usual care or intervention; the intervention is an electronic health record (EHR) alert that prompts clinicians to switch their order from normal saline to balanced crystalloids (Figure 1). PRECISE is an EHR integrated trial, which means that the entire trial workflow, from precision subphenotyping to clinician alert, is automated within Epic, our healthcare system’s EHR. The PRECISE technical implementation involved embedding the following into the Epic architecture: (1) trial inclusion criteria, (2) sepsis subphenotyping algorithm, (3) randomization, and (4) medication alert. This case study provides an in-depth technical overview as a framework for other healthcare systems to utilize EHR-integrated RCTs of ML algorithms within Epic.

## Methods

The PRECISE trial was approved by the Emory University Institutional Review Board with a waiver of informed consent due to minimal risk and the impracticability of obtaining consent in emergency fluid resuscitation settings. The clinical trial was registered on clinicaltrials.gov (NCT06253585).

**Figure 2.**
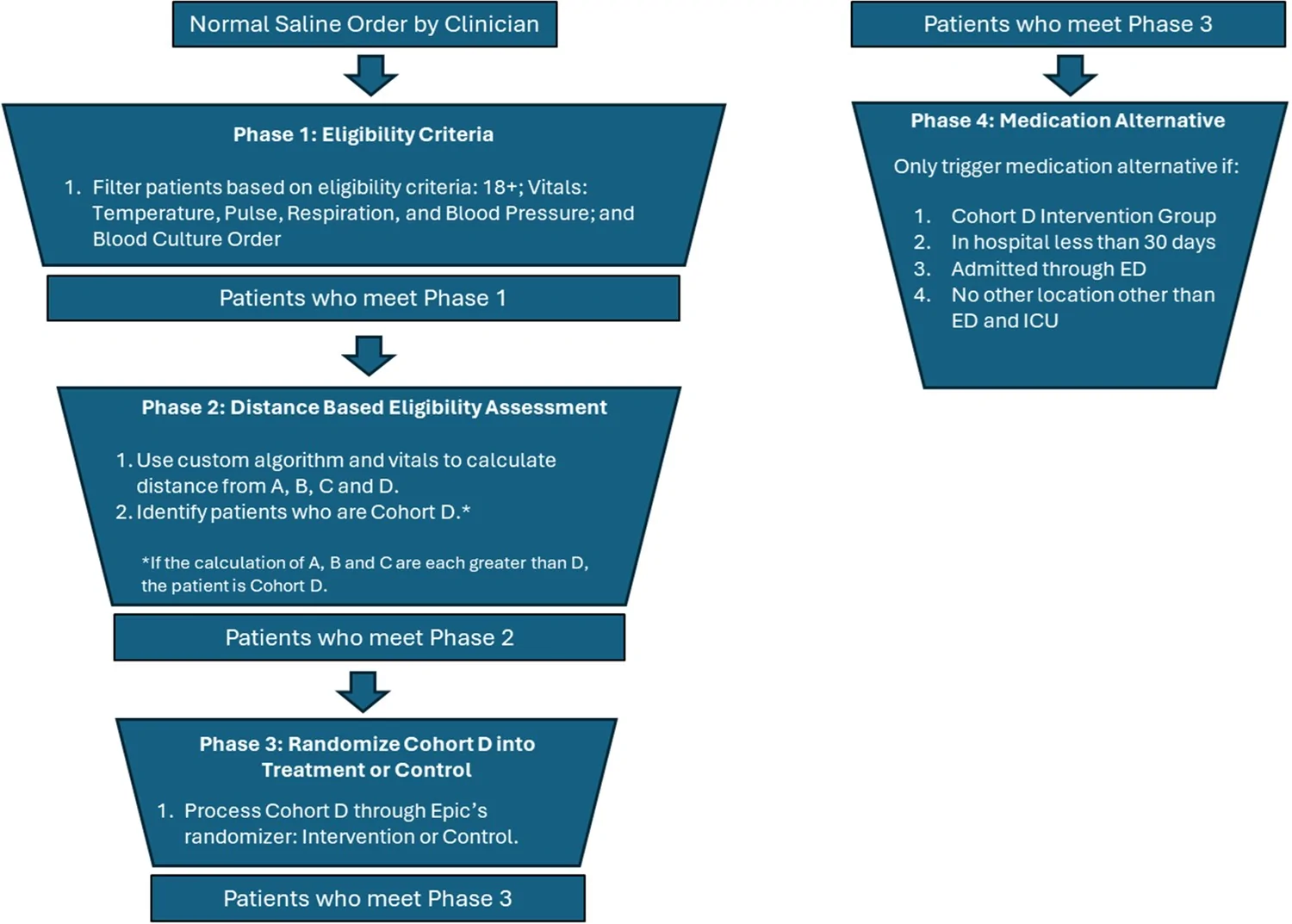
The PRECISE trial was implemented through 4 phases including building the eligibility alert, distance-based eligibility assessment, randomization process, and the medication alternative.

The trial leverages Epic functionalities through a multi-phase algorithmic approach to generate patient scores (Figure 2). We have separated the approach into 4 phases, and within each phase we provide a high-level clinical overview for the clinician/researcher and a detailed technical step-by-step process for the Epic clinician builder.

### Phase 1: initial screening

#### Clinical overview

The goal of initial screening is to evaluate for the following inclusion criteria: (1) patient is ≥18 years, (2) documentation of 1 complete set of vital signs (heart rate, temperature, respiratory rate, and blood pressure), and (3) suspicion of infection, as evidenced by blood culture order in the emergency department. While the rules are presented in the order of screening, subphenotyping, randomization, and medication alternative, the trigger to initiate this process of events was normal saline order by clinician. Thus, every time normal saline was ordered within our healthcare system, the screening process would commence.

#### Technical build

Step 1: create an acuity rule (HDA) that provides the structure to enable the rules that determine if the patient encounter meets trial criteria.

**Table:** 

Purpose	Acuity rule logic
Provides structure to enable the rules that determine if the patient encounter meets trial criteria.	First true line will be sum of the true lines will be result.

Step 2: add criteria for each inclusion or exclusion rule. Each criteria is a CER Record or rule that evaluates whether or not the criteria is true and performs an action based on the result. CER rules are records in the CER masterfile that evaluate as Boolean expressions (true or false); each rule’s criteria consist of property, operator, and value, and the rule’s context is determined by which properties are needed and where the rule can be used.

**Table:** 

Purpose	Property	Operator	Value	Result
Exclude patients younger than 18 years old	Age: years	<	18	Quit
Patient encounter has a documented temperature	Flowsheet: value for flowsheet row: temperature and return type: first value. Encounters to search: current encounter; lookback start: arrival and lookback end: discharge. Data type: single flowsheet value	=	Null	Quit
Patient encounter has a documented pulse	Flowsheet: value for flowsheet row: pulse and return type: first value. encounters to search: current encounter; lookback start: arrival and lookback end: discharge; data type: single flowsheet value	=	Null	Quit
Patient encounter has documented respirations	Flowsheet: value for flowsheet row: respirations and return type: first value. Encounters to search: current encounter; lookback start: arrival and lookback end: discharge. Data type: single flowsheet value	=	Null	Quit
Patient encounter has a documented systolic blood pressure	Flowsheet: value for flowsheet row: blood pressure and return type: first value. Encounters to search: current encounter; lookback start: arrival and lookback end: discharge. Data extensions: IP flowsheet value systolic blood pressure value only. Data type: single flowsheet value.	=	Null	Quit
Patient encounter has a documented diastolic blood pressure	Flowsheet: value for flowsheet row: blood pressure and return type: first value. Encounters to search: current encounter; lookback start: arrival and lookback end: discharge. Data extensions: IP flowsheet value diastolic blood pressure value only. Data type: single flowsheet value.	=	Null	Quit
Patient encounter fulfills all previous criteria	Always 1.	=	1	1

If all rules are true, the patient encounter will receive a score of 1 and will proceed to phase 2: distance-based eligibility assessment beginning with the subphenotyping algorithm.

### Phase 2: subphenotyping algorithm

#### Clinical overview

For patients meeting initial inclusion criteria, the subphenotyping algorithm evaluates whether the patient belongs to Group D. The subphenotyping algorithm was operationalized in Epic by defining each subphenotype as a 5D centroid (temperature, heart rate, respiratory rate, systolic blood pressure, and diastolic blood pressure). While the full algorithm centroids are not presented in step 1, the general template for the distance algorithm is presented. Through these rules, patients are assigned to Group D if they are the least distance from the Group D centroid compared to the other subphenotypes. In the calculations, erroneous non-numeric documentation of vital signs would be considered null values. However, erroneous, non-physiological, but still numeric entries would not be flagged, and would serve as inputs to the model.

#### Technical build

Step 1: create a rule (CER) to calculate Distance A using algorithm.

**Table:** 

Purpose	Context	Patient score rule logic
Calculate Distance A using algorithm	Patient score rule	Sum of true lines First true line is Distance A algorithm


$$\text{Distance A}={\left(\frac{s1\;-\;99.01216}{1.615407}-\;0.77962\right)}^{2}+{\left(\frac{s2\;-\;92.80737}{18.96842}-\;1.26826\right)}^{2}+{\left(\frac{s3\;-\;19.05414}{3.434155}-\;0.31926\right)}^{2}+{\left(\frac{s4\;-\;126.5398}{25.39245}+\;0.08937\right)}^{2}+{\left(\frac{s5\;-\;68.31196}{14.8093}-\;0.14159\right)}^{2}$$

s1—temperature; s2—heart rate; s3—respiratory rate; s4—systolic blood pressure; s5—diastolic blood pressure; each vital sign is centered and the difference between the standardized vital sign and Group A centroids are squared and summed.

Step 2: add criteria (CER) for each flowsheet row aligned with variables in algorithm.

**Table:** 

Purpose	Property	Operator	Value	Result
Pull the temperature for the Distance A algorithm	Flowsheet: value for flowsheet row: temperature and return type: first value. encounters to search: current encounter; lookback start: arrival and lookback end: discharge. Data type: single flowsheet value.	≠	−1	Property column value
Pull the pulse for the Distance A algorithm	Flowsheet: value for flowsheet row: pulse and return type: first value. Encounters to search: current encounter; lookback start: arrival and lookback end: discharge; data type: single flowsheet value	≠	−1	Property column value
Pull the respirations for the Distance A algorithm	Flowsheet: value for flowsheet row: respirations and return type: first value. encounters to search: current encounter; lookback start: arrival and lookback end: discharge; data type: single flowsheet value	≠	−1	Property column value
Pull the systolic blood pressure for the Distance A algorithm	Flowsheet: value for flowsheet row: Blood Pressure and return type: first value. encounters to search: current encounter; lookback start: arrival and lookback end: discharge; Data extensions: ip flowsheet value systolic blood pressure value only; Data type: single flowsheet value	≠	−1	Property column value
Pull the diastolic blood pressure for the Distance A algorithm	Flowsheet: value for flowsheet row: blood pressure and return type: first value. encounters to search: current encounter; lookback start: arrival and lookback end: discharge; data extensions: IP flowsheet value diastolic blood pressure value only; Data type: single flowsheet value	≠	−1	Property column value

Step 3: repeat steps 1 and 2 for each algorithm calculation B–D.

Step 4: create a rule (CER) that determines patient cohort D or not D.

**Table:** 

Purpose	Context	Patient score rule logic
Determine if the patient falls into cohort D or not D	Patient score rule	Sum of true lines First true line is result

Step 5: add criteria (CER) to the rule to compare Distances A, B, and C to D; if patient is in cohort D, then D will be the smallest calculation.

**Table:** 

Purpose	Property	Operator	Value	Result
Compare Trial Distance A with Trial Distance D	EHC Precise Trial-Distance A	>	EHC Precise Trial-Distance D	1
Compare Trial Distance B with Trial Distance D	EHC Precise Trial-Distance B	>	EHC Precise Trial-Distance D	1
Compare Trial Distance C with Trial Distance D	EHC Precise Trial-Distance C	>	EHC Precise Trial-Distance D	1

If all 3 rules are true in step 5, the patient receives a score of 3 and is advanced to the next phase, which is to randomize patients in cohort D.

### Phase 3: randomization

#### Clinical overview

Once patients meet initial inclusion criteria and are classified by the subphenotyping algorithm as Group D, Epic’s out of the box randomization algorithm assigns patients to the control arm or to the intervention arm. Control patients are given a score of 1 and intervention patients are given a score of 2. All randomized patients are included in the study regardless of subsequent phases of care (eg, discharge directly from emergency department versus admission toward vs admission to the ICU).

#### Technical build

Step 1: create a rule (CER) to randomize cohort D into treatment or control group.

**Table:** 

Purpose	Context	Patient score rule logic
Randomize cohort D patients into treatment or control	Patient score rule	Sum of true lines first true line is result

Step 2: add criteria (CER) to the rule to randomize cohort D into treatment or control group.

**Table:** 

Purpose	Property	Operator	Value	Result
Randomize cohort D patients into treatment or control	EHC precise resuscitation D or not D	=	3	C_Random Number Generator [Min = 1; Max = 2; Integer Only = Yes]

### Phase 4: medication alternative

#### Clinical overview

The intervention is a medication alternative alert (LMA) that fires when a clinician orders normal saline in a patient who meets the following criteria: (1) randomized to intervention arm, (2) initially presented to the hospital through the emergency department, (3) currently in the emergency department or an intensive care unit without having been transferred to a general ward in between, and (4) hospital stay is 30 days or less. Of note, this intervention would not be active on fluids that were ordered outside the EHR context. For instance, if fluids were verbally ordered by a clinician in an urgent resuscitation situation, there would be no LMA or equivalent trigger.

#### Technical build

Step 1: create each inclusion rule for the medication alternative (LMA), decision support records that suggest or require clinicians to choose substitute medication(s) or procedure(s) at order entry.

**Table:** 

Purpose	Name	Logic	Property	Operator	Value
Validate patient is in intervention arm	Institution defined naming convention for rule: scoring system	AND	Get score of scoring system (from randomization)	=	2
Validate patient entered through emergency department	Institution defined naming convention for rule: last emergency department	AND	Patient encounter departments ≫ specialty	Contain any	Emergency medicine
Validate patient has not been to any unit between the emergency department and intensive care	Institution defined naming convention for rule: last acute department	AND	Patient encounter departments ≫ specialty	Contain none	Medical surgical, obstetrics, obstetrics and gynecology, hematology, hematology and oncology, hospice, behavioral health, psychiatry, addiction medicine, physical medicine and rehabilitation, oncology
Validate patient has been in the hospital less than 30 days	Institution defined naming convention for rule: lookback period	AND	ADT time since admission (use hours)	<	720

Step 2: create a patient context rule (CER) that qualifies patients for the medication alternative.

**Table:** 

Purpose	Name	Logic	Property	Operator	Value
Qualify patients for the medication alternative	Institution defined naming convention for alternative rule	AND	[Name of scoring system rule]	=	True [1]
			[Name of last emergency department rule]	=	True [1]
			[Name of last acute department rule]	=	True [1]
			[Name of lookback rule]	=	True [1]

Epic system configuration for cohort D treatment medication alternative (LMA)

Step 1: create SmartText (ETX) for the instructions that will be displayed to the user when the medication alternative is displayed. ETX refers to SmartText records used to insert blocks of text into fields.

- Name: [Institution Defined Naming Convention for Medication Alternative SmartText]
- Text:
- Restrictions/Context: MR Alternatives

**Table:** 

PRECISE trial—Treatment recommendation this patient has been identified as having a potential mortality benefit from balanced crystalloids compared to normal saline. Please switch your order to lactated ringers or plasma-lyte unless there is a strong clinical indication for normal saline. For more information on this treatment recommendation, please contact Dr. Bhavani (PI) at…

Step 2: create an alternative order smartgroup (OSQ) with the medication alternatives (ERX) that will be displayed in the medication alternative alert (LMA). ERX refers to medications master-file records used for prescribing, mapping, and ordering.

- Name: [Institution defined naming convention for medication alternative smartgroup (OSQ)]
- Medications (ERX):
  - IP Order: lactated ringers bolus, intravenous once
  - IP Order: lactated ringers infusion, intravenous continuous
  - IP Order: electrolyte-A (Plasmolyte-A) bolus solution, intravenous, administer over 1 hours, once
- Suppression settings:
  - Suppress drug-drug interaction checks: YES
  - Suppress duplicate med order checks: YES
  - Suppress duplicate therapy checks: YES

Step 3: create an LMA record with the following details.

- Name: [Institution defined naming convention for LMA records]
- Type: Medication
- Smart Text (ETX): [Name of SmartText defined in step 1]
- Alternative order SmartGroup (OSQ): [Name of SmartGroup defined in step 2]
- Enable provider LMA requirements:
  - Enable ‘Allow continuation with original selection’
  - Enable ‘Require reason with original selection’

Step 4: edit the medication master file (ERX) which dictates the facility and medication(s) the LMA will fire.

- Select medication (eg, sodium chloride 0.9% intravenous solution)
- Use alternative: YES
- Add each facility and link the Precise Resuscitation Alternative Rule (CER) (as defined in epic system configuration for cohort d treatment) and LMA Record.

Initially, instead of an LMA, we had developed an “our practice advisory” (OPA) for phase 4. However, the OPA would fire with normal saline orders embedded within standardized order sets. This technical limitation meant the system would generate alerts for normal saline orders that were components of established clinical protocols, potentially leading to alert fatigue and reduced clinician compliance with the decision support tool. Thus, we chose to use the LMA instead.

Tracking of study patients was operationalized through daily extraction of cached scores, which included (1) timestamp of randomization, (2) result of randomization (0—not randomized if the patient was not Group D, 1—randomized to usual care, 2—randomized to intervention), and (3) patient identifier. The cached scores were recorded at the encounter-level and stored for subsequent linking with inpatient data.

## Results

We present the results of validation testing to evaluate the PRECISE trial build. Prior to trial initiation, the accuracy of the subphenotyping algorithm to classify Group D patients was evaluated through a total of 25 randomly selected cases with the algorithm in silent mode to identify a minimum of 5 patients in each of the 4 groups. The classification for each of these test patients was calculated off-platform and was compared to the on-platform calculation through the subphenotyping algorithm. Through this process, we found that when vital sign data were missing (*n* = 4 cases), the EHR backend defaulted to imputing values of 0, allowing group classification to proceed despite absent inputs. All other cases were accurately classified. To ensure imputation didn’t happen in the clinical trial, we added additional CER rules to phase 1 step 2, where missing vital sign values would result in non-evaluation by the screening rule. After this correction, an additional 25 chart reviews were performed to ensure that the on- and off-platform algorithm computation matched, and all 25 patients were classified accurately.

Additionally, prior to trial onset, simulation test patients were created to evaluate the appropriateness of the automated alerting system. In the PRECISE trial, patients who had a blood culture order, normal saline order, and who were classified as Group D would result in the alert firing on every order of normal saline, as long as they were in the ED or ICU without any exposure to the wards, and it had been less than 30 days since admission. The test cases included Group D patients randomized to intervention (Test cases 1 through 5), Group D patients randomized to usual care (Test cases 6 through 10), and non-Group D patients (Test cases 11 through 15). This testing was meant to ensure Group D patients in usual care and non-Group D patients would not receive the alert. Further, the testing ensured that Group D patients in the intervention arm who had been in the wards or in the hospital longer than 30 days would not receive the alert (Test cases 3-5).

**Table:** 

Case	Group	Randomization	Description	Alert outcome
1	D	Intervention	ED	Alert fired
2	D	Intervention	ED → ICU	Alert fired
3	D	Intervention	ED → ICU → Ward	Alert did not fire
4	D	Intervention	ED → Ward → ICU	Alert did not fire
5	D	Intervention	ED → ICU (LOS >30)	Alert did not fire
6	D	Usual care	ED	Alert did not fire
7	D	Usual care	ED → ICU	Alert did not fire
8	D	Usual care	ED → ICU → Ward	Alert did not fire
9	D	Usual care	ED → Ward → ICU	Alert did not fire
10	D	Usual care	ED → ICU (LOS >30)	Alert did not fire
11	Not D	N/A	ED	Alert did not fire
12	Not D	N/A	ED → ICU	Alert did not fire
13	Not D	N/A	ED → ICU → Ward	Alert did not fire
14	Not D	N/A	ED → Ward → ICU	Alert did not fire
15	Not D	N/A	ED → ICU (LOS >30)	Alert did not fire

**Figure 3.**
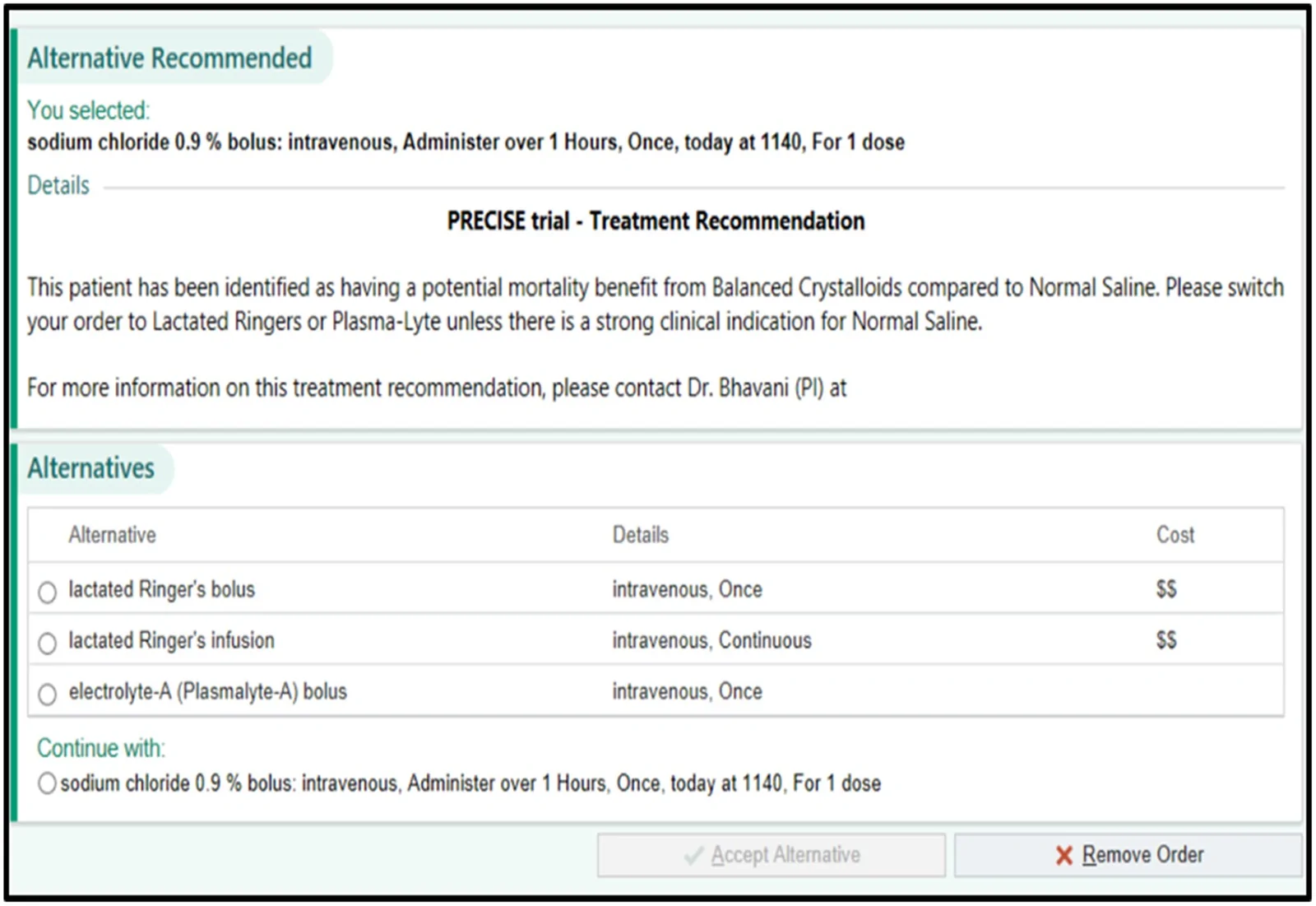
The medication alternative was designed to be minimally intrusive and maximally actionable. The alternative only fires on patients in the intervention arm of the trial when the provider has ordered normal saline. The alternative offers the balanced crystalloids to choose from, and the provider is able to stick with their original choice of normal saline if they prefer.

On June 2024, the PRECISE trial was deployed across 6 hospitals within the Emory Healthcare system, which includes academic, tertiary-care hospitals and hybrid academic-community hospitals. In total, the trial spans 6 EDs and 17 ICUs with over 300 ICU beds. The PRECISE trial was paused between October 2024 and March 2025 due to a national IV fluid shortage. In total, there were 361 522 emergency department visits screened for inclusion, with 8395 eligible patients, and 2012 classified as Group D, with 982 randomized to usual care and 1030 randomized to intervention. The PRECISE alert fired a total of 1378 times, with 26.3% of the alerts being cancelled/dismissed. The clinician-facing medication alternative that nudges the clinician to switch from normal saline to balanced crystalloids for Group D patients in the intervention arm is shown in Figure 3.

## Discussion

The PRECISE trial demonstrates the feasibility of embedding a precision medicine RCT of an ML algorithm directly within the EHR. The entire workflow, from patient identification and subphenotyping to randomization to delivery of treatment recommendation, is automated within the EHR. The approach described here is notable for: (1) building on prior EHR-integrated trials, (2) bridging the gap between ML models and clinical impact, and (3) potential generalizability to other healthcare systems and disease states beyond sepsis. This automated approach substantially reduces the personnel and operational costs that would be required to conduct a trial of this scale using traditional screening, enrollment, and intervention-delivery methods.

### Building on prior EHR-integrated trials

Over the past decade, there has been growing interest in pragmatic clinical trials embedded within the EHR. Integrating trials into the EHR enables automated patient screening, enrollment, and intervention delivery, allowing studies to scale recruitment and evaluate clinical questions that may be difficult to address using traditional trials. This capability is particularly important for time-sensitive interventions such as intravenous fluids.

EHR-integrated trials have been undertaken across a range of clinical settings. For instance, Toerper et al. conducted an EHR-integrated pragmatic emergency department trial evaluating targeted hepatitis C screening strategies.^10^ EHR-integrated trials have also incorporated randomization at the point of clinical order. For instance, Qian et al. in the ACORN trial randomized patients to 1 of 2 anti-pseudomonal therapies at the point of antibiotic order.^11^ Specifically, in the setting of fluid resuscitation, Semler et al. used an EHR-integrated trial design for providing trial information in the context of electronic order-entry system and for data collection for study patients.^5^ The PRECISE trial builds on these advances in EHR-integrated trial design but introduces an additional layer of complexity by embedding a real-time ML-based subphenotyping model to guide precision enrichment of the intervention.

### Bridging the gap between ML models and clinical impact

A major novelty of PRECISE lies in the combination of an EHR-integrated RCT with an ML model. While thousands of ML models are developed and validated in retrospective data, there is a major gap in moving from in silico to real-time implementation.^12^^,^^13^ There is an even larger gap between implementation and clinical trial testing. As of 2024, there had been only 26 RCTs of artificial intelligence (AI) algorithms in US.^14^ Prospective validation represents the most rigorous test of whether an AI model remains accurate and clinically useful when applied in real time to new patients and embedded within actual care delivery. There is a growing call to action to test AI algorithms through robust patient-centered investigations such as RCTs. Embedding trials like PRECISE directly into the EHR allows institutions to evaluate the clinical utility of ML algorithms prior to widespread implementation.

### Generalizability to other healthcare systems

A central goal of this work was to develop a framework that could be implemented in other healthcare systems. The PRECISE build leverages Epic’s native components such as acuity rules, patient score logic, randomization modules, and medication alternatives. Because the inclusion criteria, subphenotyping logic, and medication alternatives are parameterized into discrete rules, other healthcare systems that use Epic can potentially adapt these varying parts to local workflows and clinical questions.^15^ For instance, another healthcare institution interested in studying a different patient population or ML algorithm could substitute relevant parts of the build. Although PRECISE focuses on fluid type selection in sepsis, the design principles extend to any intervention where: (1) a predictive algorithm can identify a subgroup with differential treatment effects and (2) a treatment decision can be expressed as an EHR order or recommendation. Examples include antimicrobial stewardship alerts, corticosteroid initiation based on organ dysfunction trajectories, or timing of vasopressor initiation based on reinforcement learning.^16–18^

While we aim for generalizability through reporting of the trial build, there are several limitations. First, the entire trial was built using Epic functionalities, which does not generalize to institutions that use other EHR systems. Second, even for institutions using Epic, the local builds may vary. Third, the ML algorithm was decomposed into a set of linear scoring rules, with patient assignment determined by the minimum (i.e., argmin) of these scores. This process of decomposing the algorithm to fit into Epic’s simple scoring system may not be feasible for more complex models.

The technical architecture described here provides a template for other precision clinical trials embedded into the EHR. Future directions include the ability for learning health systems to incorporate EHR-embedded RCTs into the development, validation, and evaluation of ML algorithms.^19^

## Conclusion

The PRECISE trial illustrates a model for how artificial intelligence, precision medicine, and clinical trial design can converge within the EHR to test novel treatment strategies in real time. By transforming the EHR from a passive data repository into an active clinical trial platform, this approach paves the way for a new era of precision-guided research in medicine.

## Data Availability

No patient data are part of this study, but the build data from epic is presented.
